# Morphological Structure and Transcriptome Comparison of the Cytoplasmic Male Sterility Line in *Brassica napus* (SaNa-1A) Derived from Somatic Hybridization and Its Maintainer Line SaNa-1B

**DOI:** 10.3389/fpls.2016.01313

**Published:** 2016-09-02

**Authors:** Kun Du, Qier Liu, Xinyue Wu, Jinjin Jiang, Jian Wu, Yujie Fang, Aimin Li, Youping Wang

**Affiliations:** ^1^Jiangsu Provincial Key Laboratory of Crop Genetics and Physiology, Yangzhou UniversityYangzhou, China; ^2^Institute of Agricultural Sciences for Lixiahe Region in Jiangsu, Jiangsu Academy of Agricultural SciencesYangzhou, China

**Keywords:** *Brassica napus*, cytoplasmic male sterility (CMS), somatic hybridization, morphological structure, transcriptomic analysis

## Abstract

SaNa-1A is a novel cytoplasmic male sterility (CMS) line in *Brassica napus* derived from progenies of somatic hybrids between *B.*napus and *Sinapis alba*, and SaNa-1B is the corresponding maintainer line. In this study, phenotypic differences of floral organs between CMS and the maintainer lines were observed. By microscope observation in different anther developmental stages of two lines, we found the anther development in SaNa-1A was abnormal since the tetrad stage, and microspore development was ceased during the uninucleate stage. Transcriptomic sequencing for floral buds of sterile and fertile plants were conducted to elucidate gene expression and regulation caused by the alien chromosome and cytoplasm from *S. alba*. Clean tags obtained were assembled into 195,568 unigenes, and 7811 unigenes distributed in the metabolic and protein synthesis pathways were identified with significant expression differences between two libraries. We also observed that genes participating in carbon metabolism, tricarboxylic acid cycle, oxidative phosphorylation, oxidation–reduction system, pentatricopeptide repeat, and anther development were downregulated in the sterile line. Some of them are candidates for researches on the sterility mechanism in the CMS material, fertility restoration, and improvement of economic traits in the maintainer line. Further research on the tags with expressional specificity in the fertile line would be helpful to explore desirable agronomic traits from wild species of rapeseed.

## Background

Heterosis is important to improve rapeseed yield, and cytoplasmic male sterility (CMS), as one of the ideal systems for pollination control, has significantly contributed to increase rapeseed production. According to the origin of the cytoplasm, CMS in rapeseed was classified into two types (Yamagishi and Bhat, 2014). The first type was derived from mutation or intergeneric hybridization during natural reproduction, including *pol* CMS, *Shan2A*CMS, and *nap* CMS. To date, 70% of the rapeseed hybrids in China were bred using CMS lines as a pollination control system, of which *pol* CMS and *Shan2A* CMS were extensively used (Shen et al., 2008). The second type was obtained by nucleus substitution or mitochondrial gene recombination during wide hybridization or protoplast fusion between different species, including *Ogura/kos* CMS and *tour* CMS (Leino et al., 2003). As reported, CMS is a maternally inherited trait resulting from the interaction of a mitochondrial CMS gene and a nuclear fertility restoring (*Rf*) gene (Yamagishi and Bhat, 2014). For instance, the mitochondrial genome of *pol* CMS contains a causal gene named *orf224*, a chimeric gene located at upstream of and co-transcribed with the *atp6* gene. In the presence of *Rf* gene, processing of *orf224/atp6* is affected and the transcripts are specifically altered (L'Homme and Brown, 1993).

Somatic hybridization can effectively create new CMS lines by potential mitochondrial genome recombination between two parent lines. The recombination results in new open reading frames and forms chimeric genes, which affect the function of mitochondria and finally lead to the formation of CMS (Hanson and Bentolila, 2004; Carlsson et al., 2008). Previous studies reported that the mitochondrial structure of *kos* CMS and *tour*-Stiewe CMS bred from somatic hybrids differed from that of the CMS lines (*kos* and *tour* CMS) derived from the natural mutation of radish and mustard (Dieterich et al., 2003; Oshima et al., 2010). A sterile line (SaNa-1A) containing 38 chromosomes was previously selected from the BC3 progenies of *Brassica napus*–*Sinapis alba* somatic hybrids, using *B. napus* cv. “Yangyou6” as recurrent parent (Wang et al., 2005). The corresponding maintainer line (SaNa-1B), which could ensure sterility by hybridization with the CMS line, was selected by crossing SaNa-1A with different varieties of *B. napus* (Zheng et al., 2012).

Next-generation sequencing (NGS), including RNA-seq and digital gene expression (DGE), has been extensively applied in transcriptomic studies on plants. Platforms, such as Illumina HiSeq2500, Ion Torrent, and Roche 454 GS FLX+, ensure more proficient research on transcriptomes and genomes than DGEs and microarrays by improving the sequencing efficiency and reducing the costs (Liu et al., 2013; Wu et al., 2016). Recently, NGS has also been used in analyzing plant mitochondria genomes and gene expression in CMS lines. An et al. (2014) created the NIL line of *pol* CMS and analyzed the gene expression differences between floral buds of the sterile and fertile lines using RNA-seq. They identified that energy deficiency controlled by *orf224/atp6* led to the down regulation of genes regulating anther development, thus resulting in failure of sporogenous cell differentiation and pollen abortion (An et al., 2014). Yan et al. (2013) compared the gene expression differences between the young bud (<2 mm) of *Nsa* CMS and its restorer line NR1 using DGE analysis, and 11 differentially expressed genes (DEGs) uniquely expressed in the restorer line were related to the synthesis of pollen wall, including chalcone synthase gene, β-1,3-glucanase gene, and glycosyl hydrolase gene. Of these DEGs, 40% were annotated with catalytic activity, 4.4% were annotated with transporter activity, and 2.2% were annotated with antioxidant activity (Yan et al., 2013).

In the present study, we aim to compare the morphological and cytological differences between the CMS line and its maintainer line to better understand the mechanism of abortion in the CMS line. Subsequently, RNA-seq analysis was conducted on the floral bud of two rapeseed lines at the abortion stage in accordance with the cytological result. This work could be the basis for molecular dissection of the CMS mechanism in SaNa-1A.

## Materials and methods

### Plant materials

The novel CMS line (SaNa-1A) generated from progenies of somatic hybrids between *B. napus* and *S. alba* and the maintainer line (SaNa-1B) were used in this study. Plant materials were cultivated in the experimental fields of Jiangsu Institute of Agricultural Science in the Lixiahe District (Yangzhou, Jiangsu Province). Different sizes of sterile and fertile anthers were fixed for morphological analysis, aiming to acknowledge the abortion stage. In accordance with the abortion stage (tetrad stage), floral buds (0.6–1.3 mm in length) were collected and frozen at 80°C prior to semithin sections and RNA-seq.

### Semithin sections and light microscopy

Different sizes of sterile and fertile anthers were fixed in 2.5% glutaraldehyde. After 24 h, the fixative solution was replaced with a fresh solution. After 1 day, the anther was washed three times with phosphate buffer (PB; pH 7.2; 15 min for each wash) and postfixed with 1% osmium tetroxide for 4 h. The postfixed tissue was again washed three times with PB. The anther was dehydrated with a graded ethanol series (50, 70, 80, 90, 95, and 100%) for 15 min each, infiltrated with acetone, and embedded in 812 resin. Semithin sections (1 μm) were cut from the polymerized blocks and stained with 1% toluidine blue for 3 min for light microscope observations (Olympus CX51).

### RNA extraction and illumina/solexa sequencing

Total RNA from buds (0.6–1.3 mm in length, at abortion stage) of four sterile and fertile individual plants was extracted using RNAiso Plus (Takara, China) in accordance with the protocol of the manufacturer (Wang et al., 2014). The quality of total RNA was analyzed using the Agilent 2100 BioAnalyzer with threshold values of RIN ≥ 8 and 28S:18S RNA≥ 1.5:1. Then, RNA (0.1–4 μg) was precisely quantified using the QUBIT RNA Assay Kit. mRNA was purified with oligo(dT) and fragmented into 120–210 bp. cDNA was synthesized using the SuperScript II Kit (Invitrogen), following end-filling and adding A in the 3′-end. After ligation with the Illumina paired-end adapter, cDNAs were purified twice with AMPure XP Beads (Beckman) to eliminate redundant adapters and amplified with PCR. Finally, a gel purification procedure was conducted to select the fragments ranging from 300 to 350 bp to produce the paired-end library. Fragment size was controlled by the High-Sensitivity ChIP Kit, and the precise concentration of each library was tested with the KAPA qPCR Kit. A 10 μL library (2 nM) was fixed onto cBot and sequenced with Illumina HiSeq 2000.

### Processing of sequencing tags and gene expression annotation

According to Cox et al. (2010), clean reads were obtained by filtering the adapter sequences and low-quality sequences in the raw data using the FASTX toolkit (http://hannonlab.cshl.edu/fastx_toolkit), which were then assembled into contigs by the Velvet and Oases software (Zerbino and Birney, 2008). Afterwards, the clean reads were mapped onto the unigenes using Bowtie (Langmead et al., 2009). Function of unigenes was annotated by BLASTX searches against the NR, Swiss-Prot, and COG databases (*E* < 1*e*−5). The GO annotations for them were determined using Blast2GO (Conesa et al., 2005), which were then submitted to WEGO for the classification graph (Ye et al., 2006; An et al., 2014). For GO enrichment analysis, a corrected *p* ≤ 0.05 was chosen as the threshold value. The GO term (*p* ≤ 0.05) is defined as significantly enriched terms of DEGs. Pathway annotations of differential gene expression were conducted to understand gene function through BLASTX of the KEGG database. The raw reads were deposited in the NCBI Short Read Archive with the accession number SRP075203. The assembled unigenes are shown in Additional File 1.

### Analysis of DEGs

After filtering the low-quality data, all the tags were aligned to the unigenes. Similar to credibility interval approaches reported for the analysis of SAGE data (Vêncio et al., 2003), we employed IDEG6 (Romualdi et al., 2003) to identify mRNAs showing statistically significant differences based on their relative abundance (as reflected by the total count of individual sequence reads) between the two libraries. The general chi-square test was conducted, as it has been proven to be one of the most efficient tests. Finally, genes with *p* ≤ 0.01 and fold change ≥ 2 or ≤ 0.5 were marked significantly different between the two libraries.

### qRT-PCR analysis of DEGs

The DGE results were verified by real-time qRT-PCR analysis, using the same RNA samples for library construction. Two developing stages of buds (~2 mm in length, at tetrad stage; 3–5 mm in length, at or after uninucleate stage) were chosen for expression validation. First-strand cDNA was synthesized using the Revert Aid First Strand cDNA Synthesis Kit (Thermo, USA) from 1200 ng total RNA. Gene-specific primers were designed for the selected unigene sequences (Additional File 2). Reactions were conducted with the SYBR PrimeScript™ RT-PCR Kit (TaKaRa, China) in the Bio-Rad CFX96 instrument. The PCR cycling was denatured using a program of 95°C for 10 s and 40 cycles of 95°C for 5 s and 55°C for 30 s. Three biological replicates for each sample and three technical replicates were conducted, and the relative expression level was calculated using the 2^−ΔΔ*Ct*^ method. Then, *B. napus* actin was used to normalize gene expression.

## Results

### Phenotypic characterization of fertile and sterile floral buds

**Figure 1:**
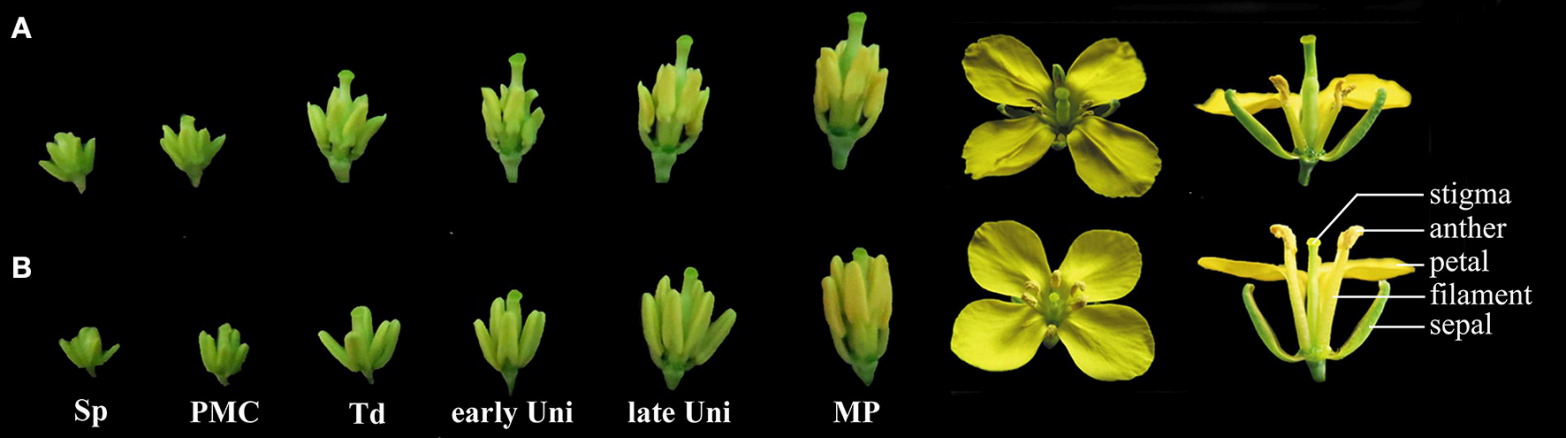
**Phenotypic characterization of fertile and sterile floral buds. (A)** Phenotype of sterile floral buds and **(B)** phenotype of fertile floral buds. Sp, sporogenous cell; PMC, pollen mother cell; Td, tetrad stage; early Uni, early uninuclear stage; late Uni, late uninuclear stage; MP, mature pollen.

The flower organ in rapeseed comprises the sepal, petal, stamen, and pistil. The male reproductive organ in normally developed rapeseed flower has four stamens longer than the other two stamens. Each stamen comprises the filament and anther, which are located at the top of each flower (Figure 1B). However, sterile flowers were visually smaller than fertile flowers, and wrinkles were clearly observed on sterile petals. During the developmental process, the anthers and filaments in sterile flowers were shorter than that in fertile flowers from the tetrad stage. The sterile anthers produced only a few or no pollen, but the pistil was normal (Figure 1A).

**Figure 2:**
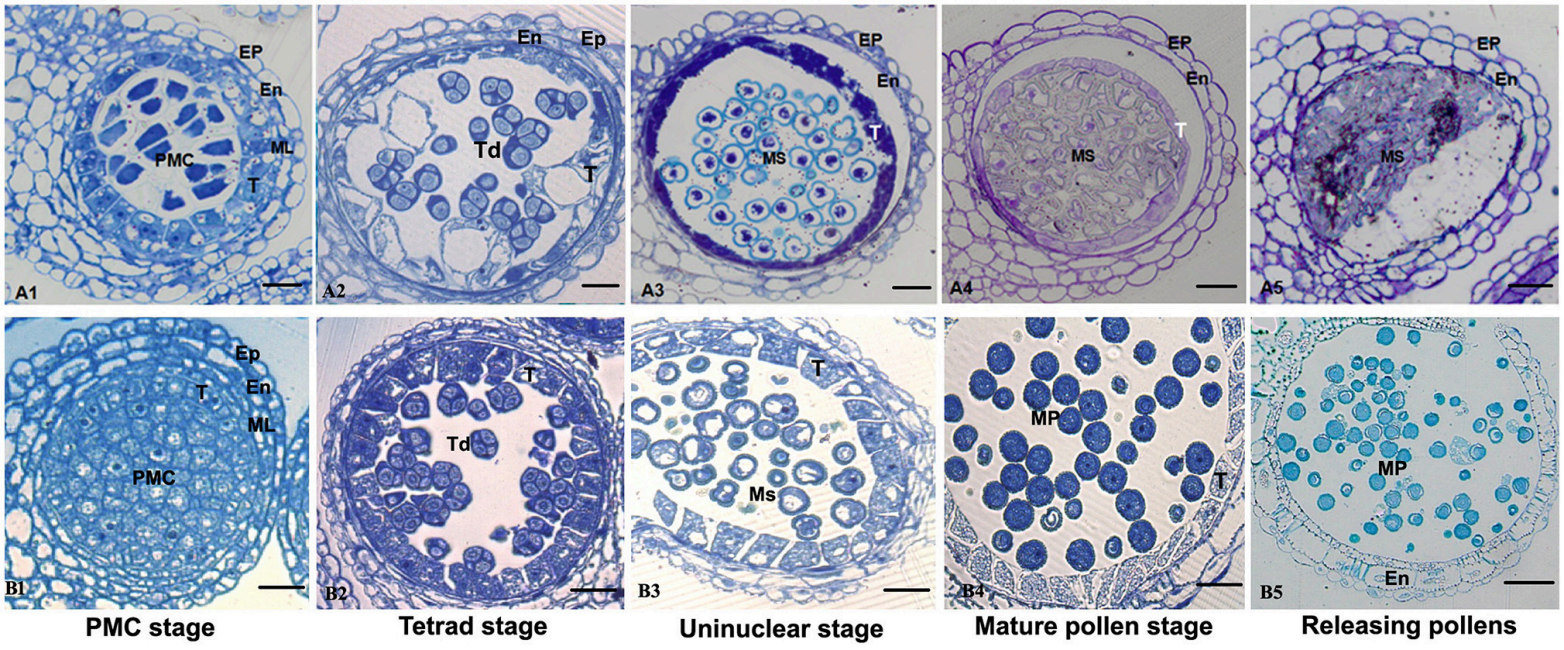
**Comparison of sterile (A1–A5) and fertile (B1–B5) anther development with toluidine blue O staining**. Bar = 10 μm for all of the stages. Ep, epidermis; En, endothecium; ML, middle layer; T, tapetum; Ms, microspore; MP, mature pollen.

Anther development in the fertile and sterile lines were cytologically observed in different developmental stages to determine the abortion stage of the sterile line. At the PMC stage, primary parietal cells were differentiated into the anther epidermis, endothecium, middle wall layers, and tapetum after periclinal division and surrounded the microspore mother cells, which were in the middle of each pollensac (Figure 2B1). At the tetrad stage, PMC cells were separated and formed tetrads after meiosis and were surrounded with callose. The middle wall layers disappeared gradually, and the tapetum cells were abundant with cytoplasm (Figure 2B2). At the uninucleate stage, the microspores were mostly occupied with central vacuole, with the nucleus squeezed to the edge, and tapetum cells started to degrade (Figure 2B3). Afterward, the microspores were differentiated into pollen grains, which were released after the breakdown of pollen sac wall (Figures 2B4,B5). Compared with the maintainer line, tapetum cells in the sterile line were observed with vacuolation during the microsporocyte and tetrad stages (Figures 2A1,A2), although they could form pollensac. In the uninucleate stage, tapetum cells were dissolved and moved to the center of the pollensac, and the cytoplasm was condensed and located at the center of microspores released from the tetrad (Figure 2A3). After the late uninucleate stage, microspores failed to separate and no mature pollen was formed, which were degraded together with tapetum cells (Figures 2A4,A5). The main reason for sterility was the obstacle in the degradation of tapetum cells, which hindered the necessary nutrition that microspores needed. We observed that the abortion of this sterile line occurred during the tetrad stage.

### Transcriptome sequencing and assembly

After removing low-quality tags in the raw data, 36,539,702 and 43,903,006 clean tags were obtained in the sterile and fertile lines, respectively (Additional File 3). A total of 195,568 unigenes (1326.8 bp on average) were obtained from clean data (average length = 96 bp) by de novo assembly with the Velvet and Oases software (Additional File 4). Considering the *N*_50_ value and average length, we select k-mer = 51 for de novo assembly. Meanwhile, 184,146 unigenes (94.2%) were equal to or greater than 200 bp and 109,168 unigenes (55.8%) were equal to or greater than 1000 bp (Table 1).

**Table 1:** **Statistic of assembly length (by Velvet and Oases software, k-mer = 51)**.

**Length**	**Number of transcripts**	**Percent (%)**
≥200	184,146	94.2
≥300	169,210	86.5
≥400	159,008	81.3
≥500	150,398	76.9
≥600	142,080	72.6
≥700	133,893	68.5
≥800	125,651	64.2
≥900	117,243	59.9
≥1000	109,168	55.8
≥1500	70,525	36.1
≥2000	41,073	21.0

### Functional annotation

**Figure 3:**
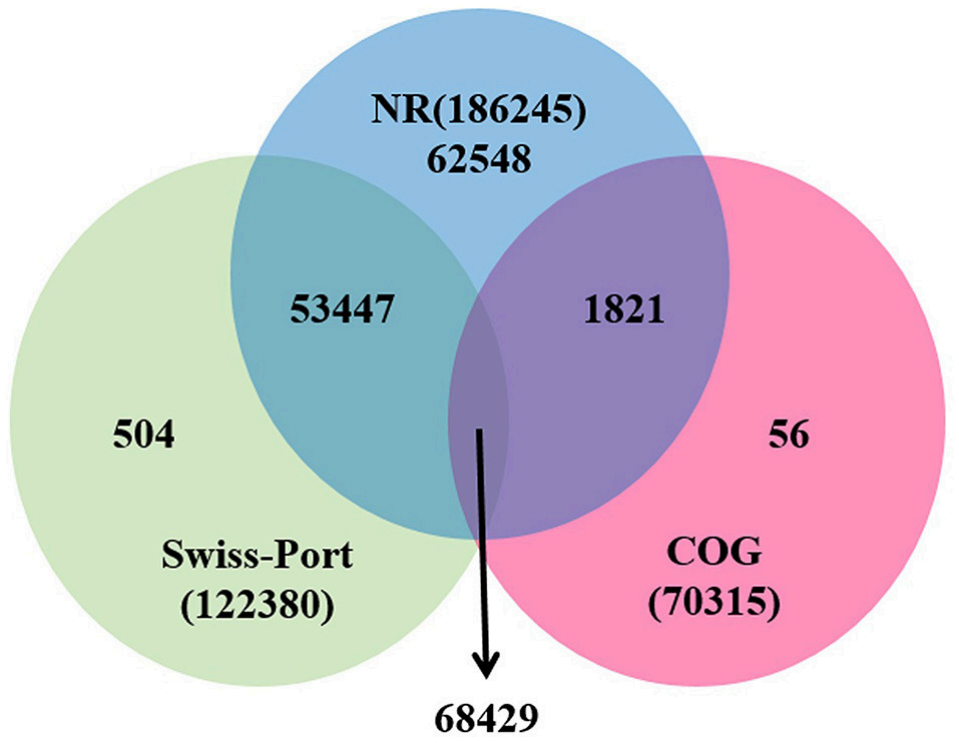
**Unigenes annotated with the public databases**. The numbers of annotated unigenes were signified in the different regions.

**Figure 4:**
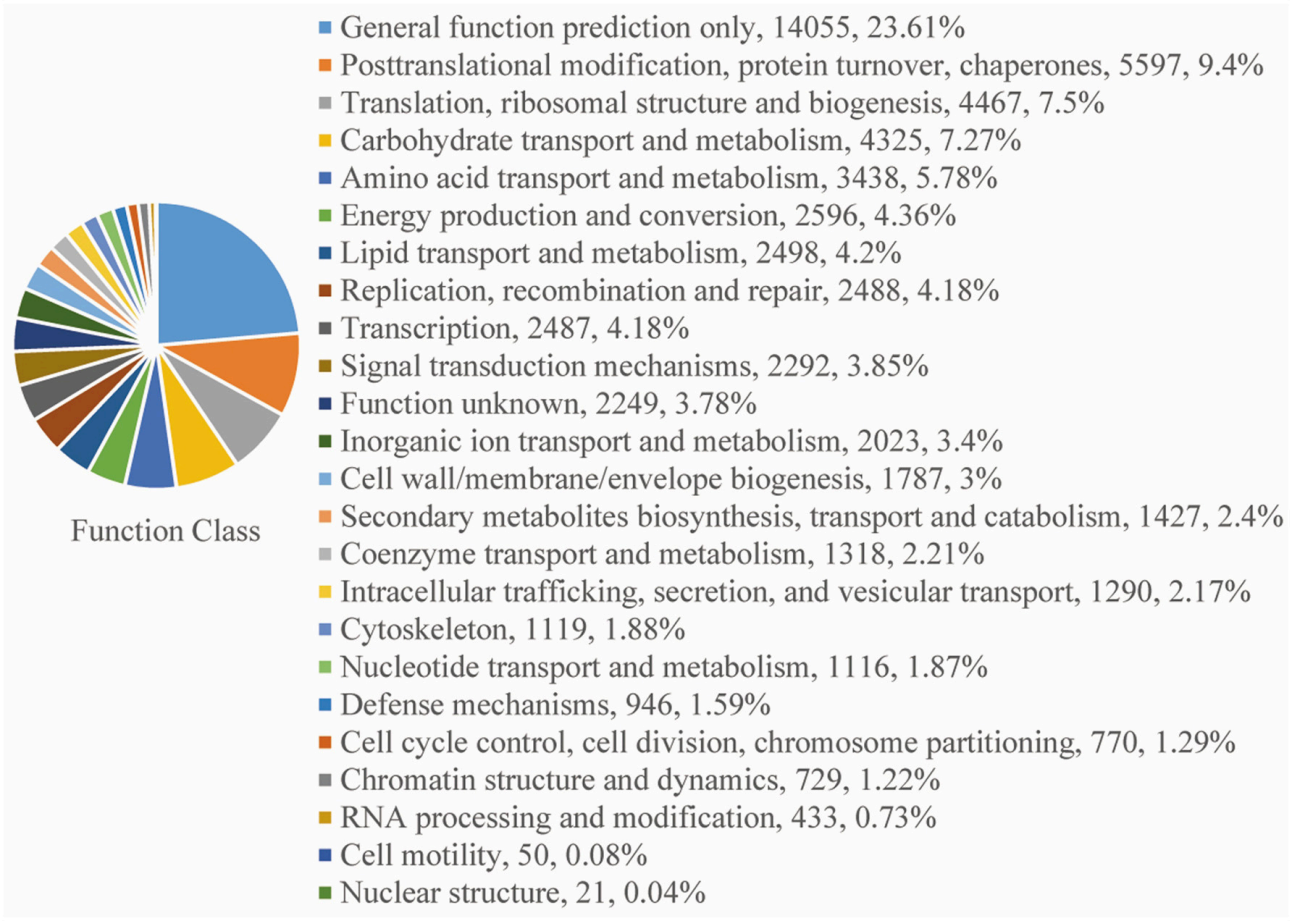
**COG functional classification**. All of the unigenes aligned in the COG database were sorted into 24 clusters.

**Figure 5:**
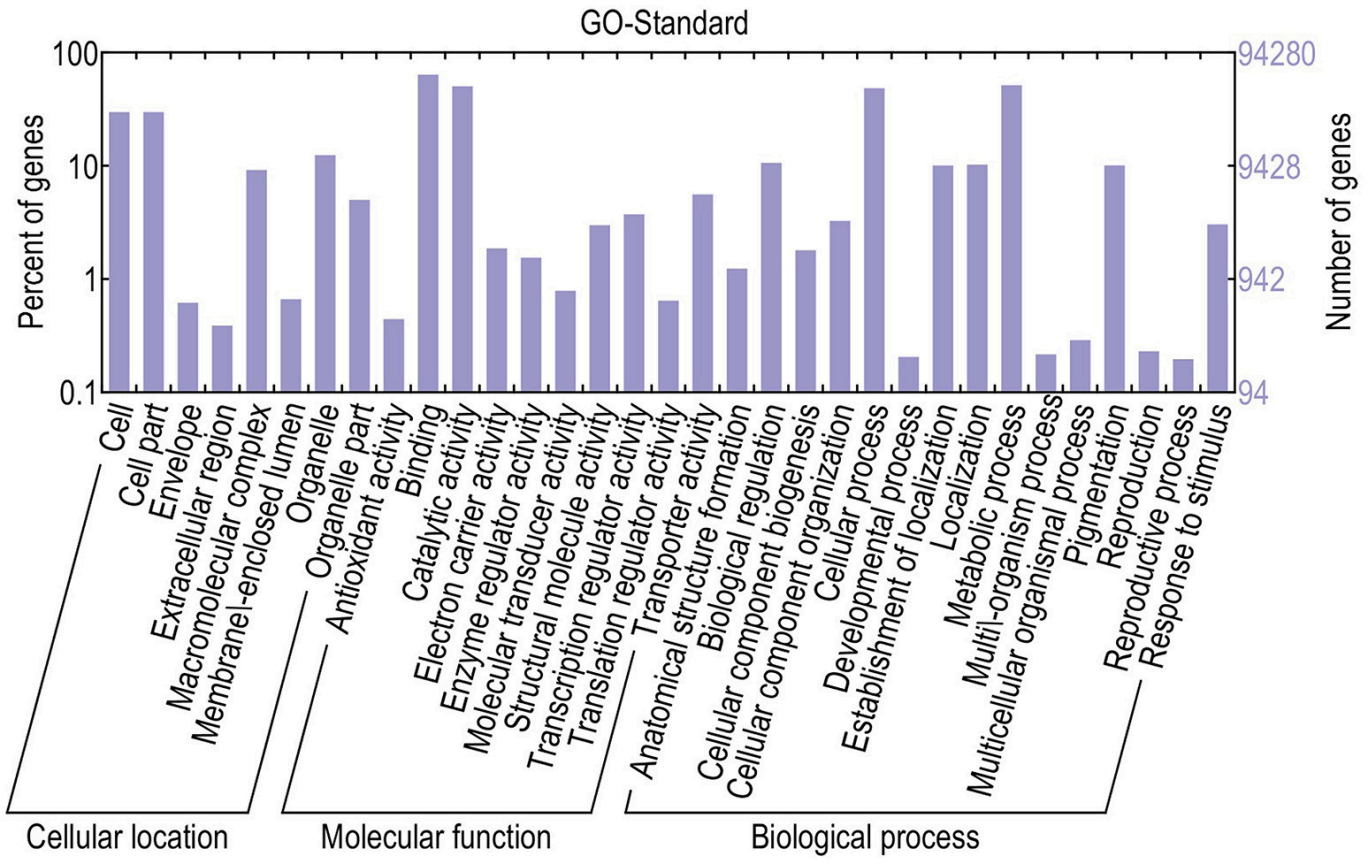
**Classification of GO annotations**. The *x*-axis indicates the subcategories; the left *y*-axis indicates the percentage of a subcategory of genes in that category; and the right *y*-axis indicates the number of unigenes in a subcategory.

For functional annotation, the 195,568 unigenes were subjected to BLASTX searches against the sequences in the Swiss-Prot, non-redundant protein sequences in the National Center for Biotechnology Information (NCBI; NR), and Clusters of Orthologous Groups (COG) databases (*E* ≤ 1*e*−5). A total of 122,380 (62.6%), 186,245 (95.2%), and 70,315 (36.0%) unigenes were annotated in the Swiss-Prot, NR, and COG databases, respectively. Meanwhile, 68,429 unigenes (35.0%) were well annotated in all of the databases and 186,752 unigenes (95.5%) were identified with annotation at least in one databases (Figure 3). In the COG database, these unigenes were classified into 24 functional groups, including general function prediction only (23.61% of unigenes), posttranslational modification, protein turnover, chaperones (9.4% of unigenes), and nuclear structure (0.04% of unigenes) (Figure 4). All the unigenes were displayed by searching GO database and classified into three hierarchies, cellular location, molecular function, and biological process. Of the 45 GO groups, unigenes were mainly classified in cell and cell part (33.90% of unigenes), binding (48.37% of unigenes), and metabolic process (33.99% of unigenes). Only a few unigenes were classified into virion, locomotion, and viral reproduction (Figure 5).

### Differential gene expression between sterile and fertile buds

**Figure 6:**
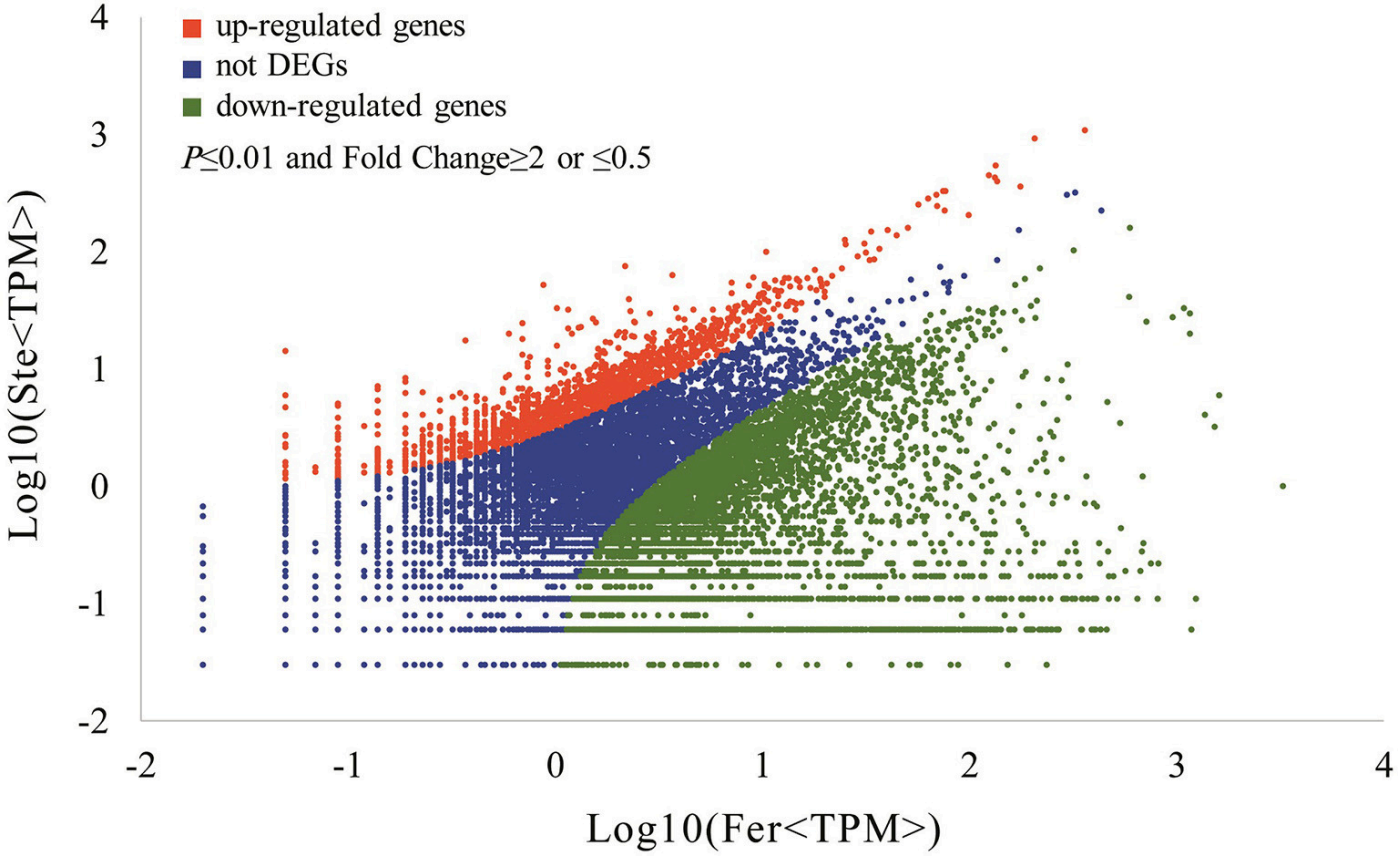
**Differentially expressed unigenes and corresponding genes in the Ste and Fer lines**. Red dots represent the upregulated transcripts in the sterile library, green dots denote the downregulated transcripts in the sterile library, and blue dots indicate the transcripts that did not change significantly. The parameters “*p* ≤ 0.01” and “fold change ≥2 or ≤0.5” were used as the threshold to evaluate the significance of gene expression difference.

The expression abundance of tag-mapped genes was analyzed by counting the number of transcripts per million (TPM). We noted that unigenes in the fertile and sterile buds showed high expression levels in several basic bioprocesses. For example, unigene304 (TPM = 663.76 in Fer and TPM = 1517.84 in Ste) encodes ribulose bisphosphate carboxylase that participates in energy production and conversion; unigene24 (TPM = 610.47 in Fer and TPM = 496.96 in Ste) encodes ubiquitin, which functions in cellular processes and signaling; and unigene78636, which is involved in RNA transport, showed high expression levels in fertile and sterile buds. With the threshold *p* ≤ 0.01 and fold change ≥2 or ≤0.5, 7811 unigenes (3.92%) were identified with expression differences between sterile and fertile lines, of which 1736 unigenes were upregulated and 6.075 unigenes were downregulated in the sterile line compared with the fertile line (Table 2 and Additional File 5). These DEGs with at least two-fold differences in the two libraries are shown in Figure 6. The red and green dots represented transcripts higher or lower in abundance for more than two-fold in the sterile library, respectively. The blue dots represented transcripts that differed less than two-fold between the two libraries, which were arbitrarily designated as “no difference in expression.”

**Table 2:** **Differentially expressed transcripts in sterile and fertile buds**.

**Class**	**Number**	**Percentage (%)**
Total transcripts	195,568	100
Expressed in Fer	193,171	98.77
Expressed in Ste	184,945	94.57
Expressed in both	182,548	93.34
Expressed only in Fer	10,623	5.43
Expressed only in Ste	2,397	1.23
Differentially expressed transcripts (*p* ≤ 0.01 and ratio ≥2 or ≤ 0.5)	Total	7811
	Up	1736
	Down	6075

### Pathway analysis and GO annotation of DEGs

**Figure 7:**
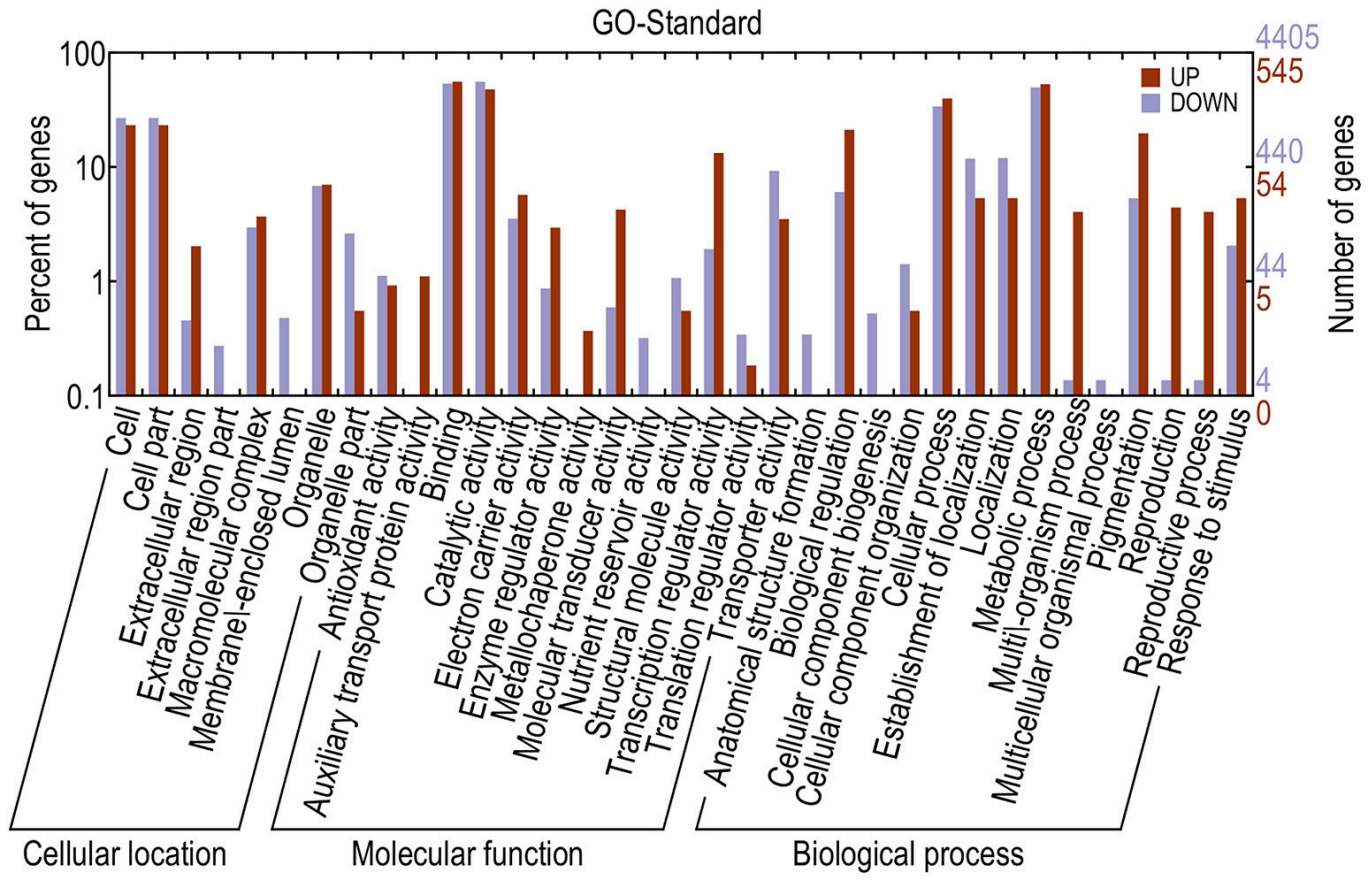
**GO annotations of DEGs**. The *x*-axis indicates the subcategories; the left *y*-axis indicates the percentage of a subcategory of genes in that category; and the right *y*-axis indicates the number of unigenes in a subcategory.

Different genes cooperate to achieve their biological functions. Pathway-based analysis helps in further understanding the biological functions of DEGs. The upregulated and downregulated unigenes were submitted to the Kyoto Encyclopedia of Genes and Genomes (KEGG) Automatics Annotation Server and classified using the single-directional best hit method (Kanehisa et al., 2008). Most of the upregulated unigenes functioned as transcription factors (TFs). By contrast, a large number of the downregulated unigenes functioned as lipid biosynthesis proteins, chaperones and folding catalysts, protein kinases, prenyltransferases, peptidases, ubiquitin systems, translation factors, and TFs (Table 3). GO annotation of DEGs revealed that, compared with the fertile line, the upregulated unigenes in the sterile line mainly functioned in the binding, cellular process, cell, and cell part. In addition, downregulated unigenes mainly functioned in the catalytic activity, binding, and metabolic process (Figure 7).

**Table 3:** **KEGG annotation of DEG unigenes**.

**Pathway**	**Total number of unigenes**	**DEGs**		
		**Total**	**Up**	**Down**
Metabolism; metabolism of terpenoids and polyketides; prenyltransferases [ko01006]	327	63	0	63
Metabolism; lipid metabolism; lipid biosynthesis proteins [ko01004]	678	85	0	85
Metabolism; glycan biosynthesis and metabolism; proteoglycans [ko00535]	13	1	0	1
Metabolism; glycan biosynthesis and metabolism; glycosyltransferases [ko01003]	1429	27	3	24
Metabolism; enzyme families; protein kinases [ko01001]	1496	70	1	69
Metabolism; enzyme families; peptidases [ko01002]	1895	61	7	54
Metabolism; enzyme families; cytochrome P450 [ko00199]	200	22	9	13
Metabolism; energy metabolism; photosynthesis proteins [ko00194]	503	5	1	4
Metabolism; amino acid metabolism; amino acid related enzymes [ko01007]	985	38	3	35
Genetic information processing; translation; translation factors [ko03012]	833	43	1	42
Genetic information processing; translation; ribosome biogenesis [ko03009]	1300	26	0	26
Genetic information processing; translation; ribosome [ko03011]	1491	18	1	17
Genetic information processing; transcription; transcription factors [ko03000]	1960	172	38	134
Genetic information processing; transcription; spliceosome [ko03041]	2149	29	1	28
Genetic information processing; replication and repair; DNA replication proteins [ko03032]	1090	13	1	12
Genetic information processing; replication and repair; DNA repair and recombination proteins [ko03400]	1586	22	10	12
Genetic information processing; replication and repair; chromosome [ko03036]	2817	64	2	62
Genetic information processing; folding, sorting and degradation; ubiquitin system [ko04121]	2003	44	1	43
Genetic information processing; folding, sorting and degradation; SNAREs [ko04131]	441	10	1	9
Genetic information processing; folding, sorting and degradation; proteasome [ko03051]	836	5	0	5
Genetic information processing; folding, sorting and degradation; chaperones and folding catalysts [ko03110]	1031	82	5	77
Environmental information processing; signaling molecules and interaction; ion channels [ko04040]	442	23	1	22
Environmental information processing; signaling molecules and interaction; GTP-binding proteins [ko04031]	598	13	3	10
Environmental information processing; signaling molecules and interaction; glycan binding proteins [ko04091]	84	14	0	14
Environmental information processing; signaling molecules and interaction; G protein-coupled receptors [ko04030]	101	2	1	1
Environmental information processing; signaling molecules and interaction; cellular antigens [ko04090]	64	3	0	3
Environmental information processing; signaling molecules and interaction; bacterial toxins [ko02042]	29	4	1	3
Environmental information processing; membrane transport; transporters [ko02000]	148	11	1	10
Environmental information processing; membrane transport; secretion system [ko02044]	299	7	0	7
Cellular processes; cell motility; cytoskeleton proteins [ko04812]	601	2	0	2

GO enrichment analysis of functional significance was conducted to reveal significantly enriched GO terms of DEGs. The GO term with *p* ≤ 0.05 was defined as significantly enriched GO term of DEGs. For molecular function, significantly enriched GO terms of DEGs included catalytic activity, ATP binding, oxidoreductase activity, transferase activity (transferring phosphorus-containing groups), and zinc ion binding (Additional File 6). For enriched biological processes, 10 GO terms of DEGs were available, which participated in the oxidation–reduction process, pollen development, transmembrane transport, carbohydrate metabolic process, cellular metabolic process, lipid metabolic process, fatty acid biosynthetic process, response to oxidative stress, cell wall modification, and cellular lipid metabolic process (Additional File 7).

### Genes related to pollen development

The development of pollen is a fundamental and complex process in flowering plants. Pollen is essential for propagation and evolution. As a model plant, *Arabidopsis thaliana* has been well investigated for this bioprocess. Therefore, all of the unigenes identified in this study were annotated to the TAIR database. Unigenes annotated to 15 genes regulating pollen development (from *AG* to *MS2*) were analysized (Higginson et al., 2003; Koizuka et al., 2003; Yang et al., 2003, 2005). One gene encoding tapetum determinant 1 (*BnTPD1*, unigene40544) was significantly upregulated in the sterile line than in the fertile line (log2ratio = 2.128). We determined that 13 unigenes were downregulated in the sterile line, including *BnAG* (unigene560, log2ratio = −5.517), *BnNZZ/SPL* (unigene15428, log2ratio = −1.951), *BnEMS1* (unigene4387, log2ratio = −2.569), *BnSERK1* (unigene11458, log2ratio = −2.206), *BnDYT1* (unigene31429, log2ratio = −3.135), *BnMYB99* (unigene2438, log2ratio = −7.081), and *BnMYB26* (unigene14326, log2ratio = −2.147). These genes mainly participated in the formation and differentiation of tapetum. Moreover, *BnTDF1* (unigene5833, log2ratio = −5.56), *BnAMS* (unigene472, log2ratio = −6.74), *BnMYB103* (unigene25542, log2ratio = −6.639), *BnMS1* (unigene17214, log2ratio = −5.98), *BnMS2* (unigene1036, log2ratio = −8.279), and *BnA6* (unigene90, log2ratio = 8.251) relate to the functional regulation of tapetum at the later developmental stage, such as degradation of callose and formation of pollen wall. We also identified unigenes with no significant expressional difference, and several other genes were unidentified in the sterile and fertile lines (Additional File 8).

### Genes involved in carbon metabolism

Accumulation of starch is necessary for the development of microspore. In other words, abundance of starch is an important characteristic of fertile pollen. Genes participated in carbon metabolism were differentially expressed between fertile and sterile lines. Altered expression of numerous genes involved in pentose and glucuronate interconversions, starch and sucrose metabolism, and amino sugar and nucleotide sugar metabolism were observed (Additional File 5). With regard to pentose and glucuronate interconversions, 13 genes were significantly downregulated, including 6 genes encoding aldehyde dehydrogenase (unigene242, unigene1535, unigene2791, unigene5839, unigene7055, and unigene7131), 4 genes encoding pectinesterase family protein (unigene7776, unigene8408, unigene17104, and unigene25928), and 3 genes encoding pectate lyase family protein (unigene13148, unigene15151, and unigene31020). Moreover, 9 genes related to starch and sucrose metabolism were significantly downregulated, including 5 genes encoding pfkB-type carbohydrate kinase family protein (unigene981, unigene2366, unigene4077, unigene16521, and unigene20485), 1 gene encoding beta-glucosidase (unigene4620), 2 genes encoding glycosyl hydrolase (unigene13516 and unigene17104), and 1 gene encoding cell wall invertase (unigene13728). Of the genes mentioned above, unigene8408, unigene17104 and unigene13148 were not identified in sterile line. Two genes participating in amino sugar and nucleotide sugar metabolism were downregulated, encoding mannose-6-phosphate isomerase (unigene25396, log2ratio = −2.859) and 6-phosphofructokinase (unigene28654, log2ratio = −2.907), respectively.

### DEGs involved in citrate cycle [tricarboxylic acid (TCA) cycle] and oxidative phosphorylation

The mitochondria is an important site for numerous metabolic pathways, including the TCA cycle, respiratory electron transfer, and ATP synthesis (Barrientos, 2003; Reichert and Neupert, 2004; Logan, 2006). The recombination and rearrangement of mitochondrial genomes could cause dysfunction and lead to CMS. Flower phenotypic variation or defection in pollen formation are presumed as secondary effects of mitochondrial mutation, and the primary defect may be a reduction in the efficiency of respiration or the impairment of other mitochondrial functions. We determined that six unigenes participating in the TCA cycle were significantly downregulated, with expressions increased by 3.43–53.47-folds in Fer, such as unigene12291 encoding a subunit of aconitate hydratase, unigene5423 encoding a subunit of citrate synthase, unigene232 encoding phosphoenolpyruvate carboxylase 2, unigene2462 encoding peroxisomal nad-malate dehydrogenase 1, unigene579 encoding 2-oxoglutarate dehydrogenase E1 component, and unigene40435 encoding the subunit B2 of ATP citrate lyase (Additional File 5). Citrate synthase, as a key enzyme of the TCA cycle, functions in the formation of citrate using acetyl-CoA and oxaloacetic acid as substrate. We also identified 11 DEGs related to the electron transport chain and oxidative phosphorylation, which were downregulated in the sterile line compared with the fertile line. For instance, four genes (unigene1243, unigene5691, unigene46531, and unigene31126) encoding subunits of ATPase, three genes (unigene7780, unigene56318, and unigene27486) encoding subunits of pyrophosphorylase, unigene26033 encoding subunits of cytochrome oxidase, unigene15953 and unigene952 encoding NADH dehydrogenase, and unigene1560 encoding subunit of ubiquinol oxidase were included (Additional File 9).

### Genes involved in oxidoreductase activity

In the present study, oxidoreductase activity is one of the most enriched GO terms. A total of 145 DEGs were involved in oxidoreductase activity, including 36 members acting on the aldehyde or oxo group of donors, with NAD or NADP as acceptor. For instance, fatty acyl-CoA reductase (alcohol-forming)/oxidoreductase (unigene1036 and unigene7520), also called male sterility 2 (MS2), acting on the CH–CH group of donors, with NAD or NADP as acceptor, was more abundant in the fertile line. Other DEGs were 3-chloroallyl aldehyde dehydrogenase/aldehyde dehydrogenase (unigene1535, unigene5839, and unigene7055), aldehyde oxidase (unigene3230, log2ratio = −2.686), NAD or NADH binding/catalytic/glyceraldehyde-3-phosphate dehydrogenase (unigene1766, log2ratio = −1.898), cytosolic factor family protein (unigene12736 and unigene27821), and several unnamed oxidoreductase members (Additional File 5).

### Differentially expressed pentatricopeptide repeat (PPR) proteins

Proteins encoding the PPR motif are predicted as site-specific, RNA-binding adaptor proteins that mediate the interactions between RNA substrates and relative enzymes (Lurin et al., 2004; Stern et al., 2004; Shikanai, 2006). PPR proteins play important role in plant mitochondrial biogenesis (Wen et al., 2003). Most of the cloned restorer genes encode mitochondria-targeted PPR proteins (Brown et al., 2003; Desloire et al., 2003; Kazama and Toriyama, 2003; Koizuka et al., 2003; Akagi et al., 2004; Wang et al., 2006). In this study, 12 genes encoding PPR proteins (unigene1911, unigene16513, unigene16881, unigene17672, unigene20560, unigene21040, unigene23771, unigene27924, unigene28403, unigene40662, unigene42447, and unigene44799) were downregulated and one gene (unigene57904) was upregulated (Additional File 5). These PPR proteins were candidates for analyzing the link between nuclear and mitochondrial gene expression.

### Confirmation of DEGs by quantitative reverse transcription polymerase chain reaction (qRT-PCR)

**Figure 8:**
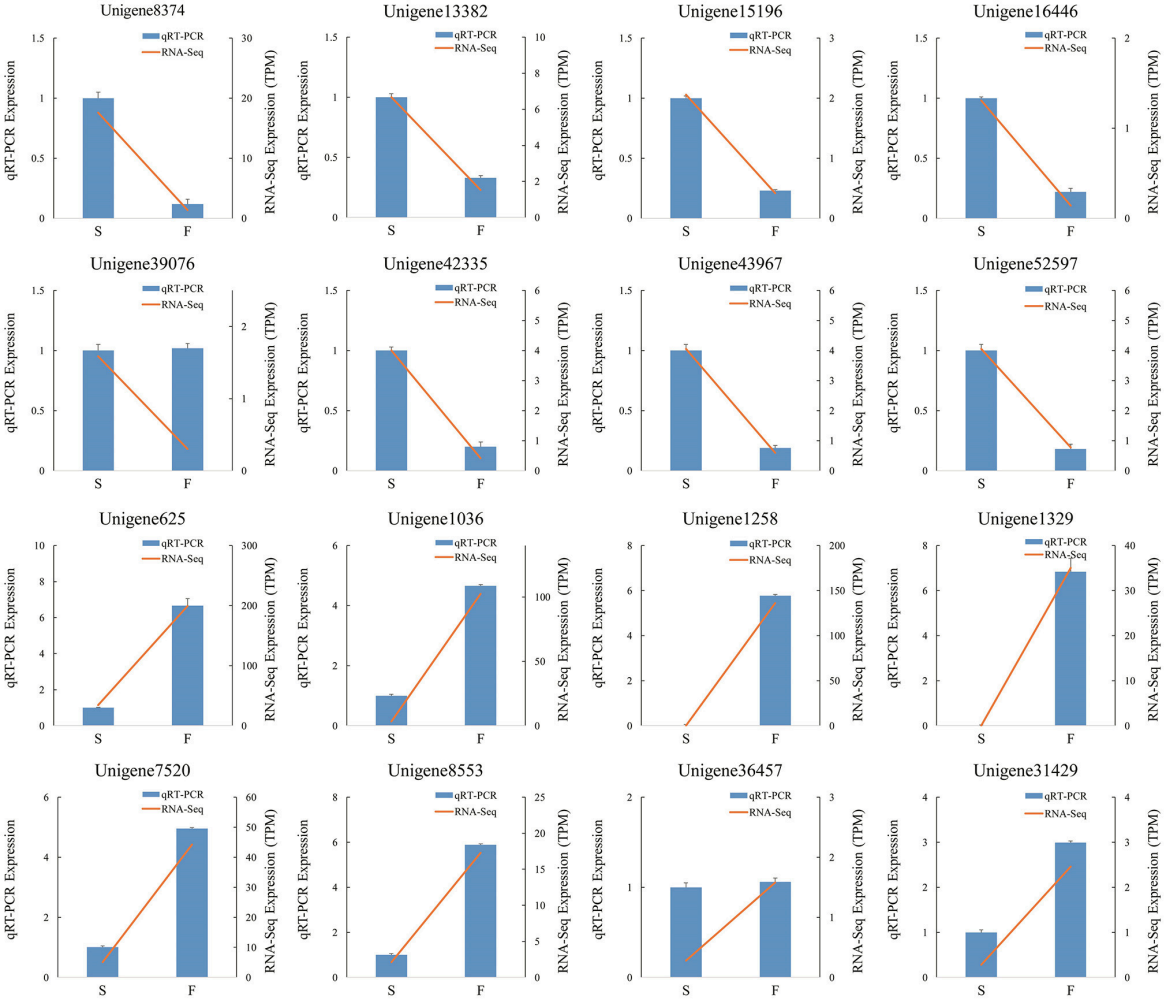
**qRT-PCR verification of differentially expressed unigenes**. S denotes the sterile sample and F denotes the fertile sample.

We randomly selected 16 genes for qRT-PCR assays to confirm the reliability of the Illumina/Solexa sequencing technology. The corresponding primers are listed in Additional File 2. The results showed that the expression of 14 genes were consistent between qRT-PCR and RNA-seq analyses (Figure 8). In qRT-PCR, unigene39076 and unigene36457 showed no difference in the fertile and sterile buds, whereas RNA-Seq analysis indicated a significant difference. The inconsistency between qRT-PCR and RNA-seq analyses of certain genes can be likely attributable to the fact that RNA-seq was more sensitive in the detection of low-abundance transcripts and small expressional changes than qRT-PCR.

## Discussion

Some considerable differences from the morphology of floral organs between sterile and fertile lines were observed. And by comparing different anther developmental stages between the two lines through microscope observation, we found that anther development in SaNa-1A was abnormal since the tetrad stage, and the development of microspores ceased during the uninucleate stage.

Subsequently, the transcriptome of fertile and sterile buds in rapeseed were acquired using the Illumina sequencing. A total of 195,568 unigenes were obtained, and 4% of them (7811 unigenes) were identified with significant expression differences, indicating that, although the bud development is a complicated and polygenic process, expressional changes in a small number of genes can alter the trait observably. All the DEGs could be categorized into 35 classifications by GO functional analysis, which were involved in biochemistry, metabolism, growth, development and so on (Figure 7). For enriched biological processes, ten GO terms of DEGs were involved in oxidation–reduction process, pollen development, transmembrane transport, carbohydrate metabolic process, cellular metabolic process, lipid metabolic process, and so on (Additional File 7). These DEGs would be candidates for further research.

Pollen formation in flowering plants depends on the differentiation and interaction of the reproductive cells named microsporocytes and the somatic cells that form the tapetum. The microsporocytes generate microspores, and the tapetum cells support the nutrition for development of mature pollen grains. A comparison of morphological structure during anther development revealed that the tapetum in the sterile line was vacuolated during the tetrad stage. Moreover, microspore development was aberrant in the sterile line during the uninucleate stage compared with the fertile line, resulting in pollen abortion. The *TPD1* gene is necessary for tapetum cell specialization in the *Arabidopsis* anther (Yang et al., 2003). In male sterile mutant *tpd1*, the precursors of tapetum cells were differentiated and developed into microsporocytes instead of tapetum. Ectopic overexpression of *TPD1* in the wild-type *Arabidopsis* carpel caused a significant increase in the number of carpel cells. Furthermore, the activation of cell division in the transgenic carpel by *TPD1* overexpression was dependent on *EMS1*/*EXS* (Yang et al., 2005). *AtMYB103*, as a member of the *R2R3 MYB* family, is uniquely expressed in the tapetum and anther. In the transgenic line with downregulated *AtMYB103*, the shape of the pollen was distorted, the content of the cytoplasm was reduced or no cytoplasm could be observed, and early degeneration of the tapetum occurred (Higginson et al., 2003). Moreover, *AMS* acts as a master regulator coordinating pollen wall development and sporopollenin biosynthesis in *Arabidopsis* (Xu et al., 2014). In the present study, we observed that the expression of *BnAG, BnSPL/NZZ, BnEMS1, BnAG*, and *BnDYT1* was significantly downregulated in the sterile lines, which might cause abnormal tapetum development in the sterile line. The observed downregulation of *BnAMS, BnMYB103, BnMS1*, and *BnMS2* in the sterile line could directly block the synthesis of sporopollenin in the pollen wall. It has been reported that nuclear-mitochondrial interaction results in CMS in previous studies (Chase, 2007; Jing et al., 2012). These genes were downregulated, which consequently led to the cease of microspores development in the sterile buds.

Rearrangements of mitochondria genome could alter the expression of genes involved in respiration and ATP synthesis, affecting ATP formation and physiological processes in mitochondria (Bergman et al., 2000). Depression in ATP production or carbohydrate accumulation has been identified by other researchers in “late stage” CMS flowers (Datta et al., 2002). In this study, six DEGs involved in the TCA cycle were downregulated in the sterile line. DEGs involved in respiration/ATP synthesis and oxidoreductase activity were also identified. And 11 DEGs were involved in the oxidative phosphorylation pathway, which affects NADH dehydrogenase, pyrophosphorylase, and ATP synthase, and these genes were downregulated in the sterile line. Oxidoreductase activity was one of the most enriched GO terms, whereas oxidative stress during microsporogenesis was assumed to induce premature abortion of tapetum cells because of programmed cell death in CMS lines of sunflower and rice (Balk and Leaver, 2001; Chinnery, 2003; Li et al., 2004). Expressional alternation of numerous genes involved in pentose and glucuronate interconversions, starch and sucrose metabolism, and amino sugar and nucleotide sugar metabolism were also observed. The nature of mitochondrial genes influencing the expression of nuclear genes was unclear. Further investigations on them will help illuminating the primary targets and downstream components of CMS-associated mitochondrial signaling pathways in the sterile line.

Nuclear genes are important to control the biosynthesis and function of mitochondria (Andersson et al., 2003). Approximately 10% of eukaryotic nuclear genes encode proteins that are targeted to mitochondria following the synthesis of cytosolic ribosomes (Fisk et al., 1999). The majority of PPR proteins are targeted to plastid or mitochondria (Nakamura et al., 2003), and many genetic and biochemical studies conclude that PPRs directly bind to a specific RNA sequence and promote anterograde regulation, such as posttranscriptional splicing, processing, editing, or regulating mRNA stability (Kotera et al., 2005; Schmitz-Linneweber et al., 2005; Okuda et al., 2006, 2007; Hattori et al., 2007; de Longevialle et al., 2007). To date, 60% of 450 PPR proteins in *Arabidopsis* were predicted to be related to mitochondria. PPR motifs have been proposed to possess binding properties to proteins and RNA (Small and Peeters, 2000; Williams and Barkan, 2003). Mutation of these genes in humans is accompanied by cytochrome *c* oxidase deficiency (Mootha et al., 2003; Shadel, 2004). PPR proteins are valuable candidates for nuclear-encoded factors controlling distinct steps of transcript maturation in mitochondria. Many of these PPR proteins may be essential to the CMS systems, such as the *Rf* gene encoding a member of the PPR (Yamagishi and Bhat, 2014). PPR proteins have also been reported as targets of different miRNAs (Rhoades et al., 2002; Sunkar and Zhu, 2004). In this study, 13 PPR proteins showed differential expression between two rapeseed lines, including 1 upregulated PPR protein and 12 downregulated PPR proteins in the sterile line. These PPR proteins are candidates for analyzing the link between nuclear and mitochondrial gene expression.

## Conclusions

In this study, we compared the transcriptome data between floral buds of the sterile and fertile lines in rapeseed using high-throughput transcriptomic sequencing. A total of 7811 genes were identified with expression differences between the 2 rapeseed lines, of which 1736 genes were upregulated and 6075 genes were downregulated in the CMS line compared with the maintainer line. GO-based and pathway-based analyses indicate that these DEGs were related to diverse molecular functions. The results provide a basis for future research on the CMS mechanism in SaNa-1A, fertility restoration, and improvement of agronomic traits. We should focus on characterizing the functions of these candidate genes.

## Author contributions

YW conceived and designed the study. KD, QL, XW, and JJ participated in the experiments. JW and YF analyzed the data. AL bred the material. All authors drafted the manuscript and approved the final manuscript.

### Conflict of interest statement

The authors declare that the research was conducted in the absence of any commercial or financial relationships that could be construed as a potential conflict of interest.

## Abbreviations

CMS: Cytoplasmic male sterility
COG: Clusters of orthologous groups
DGE: Digital gene expression
GO: Gene Ontology
KEGG: Kyoto Encyclopedia of Genes and Genomes
NGS: Next-generation sequencing
PB: Phosphate buffer
PPR: Pentatricopeptide repeat
qRT-PCR: Quantitative reverse transcription polymerase chain reaction
TCA: Tricarboxylic acid
TFs: Transcription factors
TPM: Transcripts per million.
